# Positive and negative early life experiences differentially modulate long term survival and amyloid protein levels in a mouse model of Alzheimer's disease

**DOI:** 10.18632/oncotarget.9776

**Published:** 2016-06-01

**Authors:** Sylvie L. Lesuis, Herve Maurin, Peter Borghgraef, Paul J. Lucassen, Fred Van Leuven, Harm J. Krugers

**Affiliations:** ^1^ Swammerdam Institute for Life Sciences, Center for Neuroscience, University of Amsterdam, Amsterdam, The Netherlands; ^2^ Experimental Genetics Group - LEGTEGG, Department of Human Genetics, KU Leuven, Leuven, Belgium

**Keywords:** early life stress, early handling, biAT, Alzheimer, glucocorticoids, Gerotarget

## Abstract

Stress has been implicated as a risk factor for the severity and progression of sporadic Alzheimer's disease (AD). Early life experiences determine stress responsivity in later life, and modulate age-dependent cognitive decline. Therefore, we examined whether early life experiences influence AD outcome in a bigenic mouse model which progressively develops combined tau and amyloid pathology (biAT mice).

Mice were subjected to either early life stress (ELS) or to ‘positive’ early handling (EH) postnatally (from day 2 to 9). In biAT mice, ELS significantly compromised long term survival, in contrast to EH which increased life expectancy. In 4 month old mice, ELS-reared biAT mice displayed increased hippocampal Aβ levels, while these levels were reduced in EH-reared biAT mice. No effects of ELS or EH were observed on the brain levels of APP, protein tau, or PSD-95. Dendritic morphology was moderately affected after ELS and EH in the amygdala and medial prefrontal cortex, while object recognition memory and open field performance were not affected. We conclude that despite the strong transgenic background, early life experiences significantly modulate the life expectancy of biAT mice. Parallel changes in hippocampal Aβ levels were evident, without affecting cognition of young adult biAT mice.

## INTRODUCTION

Alzheimer's disease (AD) is a neurodegenerative disorder characterised by progressive impairments in cognitive functions [1]. Prominent neuropathological features of AD are amyloid-containing plaques and neurofibrillary tauopathy consisting of threads and tangles (NFT), which are observed throughout the brain, including areas critically involved in memory formation and emotional regulation [2]. The accumulation of amyloid plaques and tauopathy is believed to underlie neuronal and synaptic dysfunction, and age-related cognitive decline [3, 4].

While genetics, in particular specific mutations, have been implicated in rare forms of familial dementia [5], the aetiology of the large majority of late-onset however, sporadic AD cases remains elusive. It has been hypothesised that gene-environment interactions, epigenetic factors, exercise, diet, stress, and life-style in general, contribute to the incidence and progression of late-onset sporadic AD [6, 7]. Epidemiological studies support a role for chronic stress as an important environmental risk factor for AD progression [8, 9]. Indeed, elderly individuals that are prone to psychological distress are more likely to develop AD than non-stressed individuals of the same age [10]. Animal model studies substantiate this role of environmental modulation of AD. Chronic stress in adulthood e.g. elevates Aβ_40_ and Aβ_42_ brain levels, accelerates amyloid plaque formation, increases tauopathy and neuronal atrophy, and impairs learning and memory in various mouse models [11–13].

Stress during the period of early life, when the brain is still developing, often has more pronounced exposure and longer lasting effects compared to adult stress. Moreover, early life stress not only enhances sensitivity of the brain to subsequent later stressors, but also accelerates age-related cognitive decline [14–19]. In contrast, daily handling (EH) during the early postnatal period is known to increase maternal care, blunts the sensitivity to stressors later in life, and reduces age-related cognitive decline in wild type rodents [14, 20].

While early life experiences influence cognition later in life, it remains elusive whether they also affect the development of AD pathology and reduce life expectancy in AD models. We therefore imposed ‘negative’ *versus* ‘positive’ experiences during the early period from postnatal day (PND) 2-9 and analysed whether they differentially influenced AD-related brain changes in transgenic APP.V717I x Tau.P301L (biAT) mice. biAT mice are well characterised to develop both amyloid plaques and neurofibrillary tau pathology over time [21]. We examined the effects of ELS and EH in BiAT mice on (i) the life expectancy, (ii) amyloid levels and synaptic proteins in the brain, (iii) behavioural performance, and (iv) the dendritic architecture in brain areas relevant for memory formation.

## RESULTS

### Body weight

**Figure 1:**
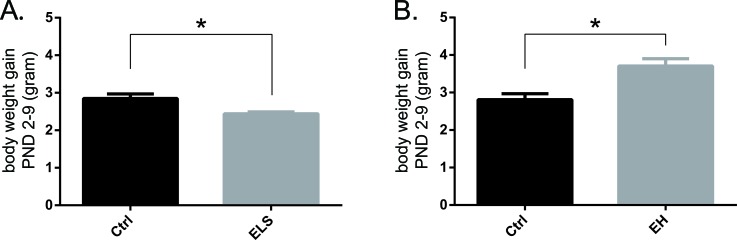
Early life experiences acutely affect body weight of biAT mice Body weight gain measured from PND 2 and 9 in **A.** ELS litters (Ctrl: *n* = 7; ELS: *n* = 7; t(11) = 2.96; *p* < 0.05), and **B.** EH animals (Ctrl: *n* = 5; EH: *n* = 4; t(4) = −2.93; *p* < 0.05) each compared to control reared biAT mice. Data are expressed as mean ± SEM.

Housing litters in a cage with limited nesting and bedding material from PND 2-9 fragments maternal care and is a well-known model to elicit early life stress (ELS) in the offspring [22, 23]. In our study, offspring of biAT mice that were stressed during early life showed a 15% reduction in body weight gain from PND 2-9, compared to control-reared litters from the same genetic background, (t(11) = 2.96; *p* < 0.05) (Figure 1A).

In another group of biAT mice, the pups were separated from the dam daily for 15 minutes (early handling, EH) in the same PND 2-9 timeframe. This is known to enhance maternal care upon reunion of the dam with her pups and is a commonly used model for early life enhancement [24, 25]. EH increased body weight gain significantly by 32% compared to control-reared mice (t(4) = −2.93; *p* < 0.05) (Figure 1B).

When reaching adulthood, these differences in body weight were no longer evident between the four groups of biAT mice (data not shown).

### Life expectancy

**Figure 2:**
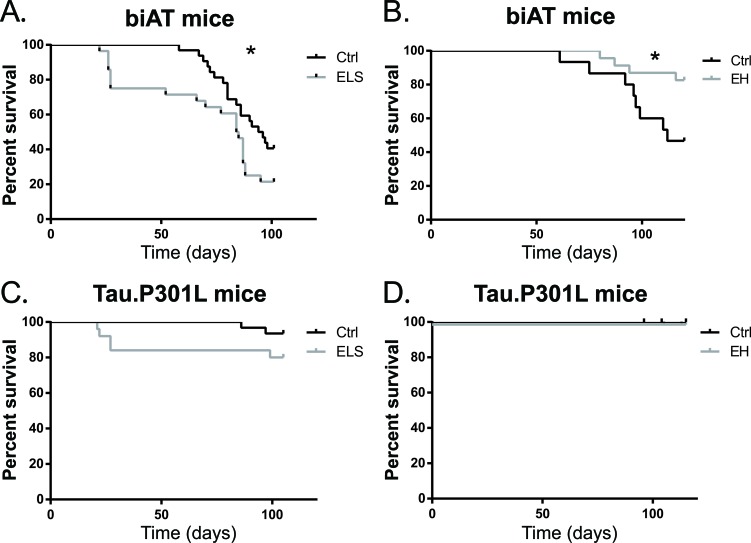
ELS exacerbates early death while EH prolongs survival of young biAT mice **A.** ELS significantly decreased survival of biAT mice (Ctrl: *n* = 32; ELS: *n* = 28; χ^2^(1) = 4.35; *p* < 0.05). **B.** EH prolonged survival compared to control mice (Ctrl: *n* = 15; EH: *n* = 23; χ^2^(1) = 4.15; *p* < 0.05). **C.** Tau.P301L littermates were not significantly affected by ELS (Ctrl: *n* = 31; ELS: *n* = 25; χ^2^(1) = 1.17, ns) or **D.** by EH (Ctrl: *n* = 17; EH: *n* = 6; χ^2^(1) = 0.62, ns).

Even biAT mice reared under control conditions, are known to suffer severe mortality between age 6-8 weeks and adulthood [21] (Figure 2A). Interestingly, the survival rate of biAT mice that were stressed early in life was significantly lower compared to control biAT mice,. The survival in the ELS-group remained significantly lower until the end of the experiment (χ^2^(1) = 4.35; *p* < 0.05). At P110, 40.3% of biAT control animals were still alive, while only 20.3% of the biAT animals that were exposed to ELS survived. Survival was not affected by ELS in Tau.P301L littermates during the same time window of this study (χ^2^(1) = 1.17, ns, Figure 2B).

In the EH-experiment we observed a similar early mortality of the biAT control animals (Figure 2C). Most interestingly, in contrast to ELS, the EH treatment significantly increased the life expectancy of biAT mice: 82.6% were still alive at PND 110 compared to only 46.7% of the control biAT animals (χ^2^(1) = 4.15; *p* < 0.05) (Figure 2C). Again, survival of the Tau.P301L littermates undergoing exactly the same treatment was not significantly affected (χ^2^(1) = 0.62, ns; Figure 2D). Given the strong effects of these early life experiences on life expectancy of the biAT mice, as opposed to the Tau.P301L littermates, all subsequent experiments were conducted in biAT mice.

### Hippocampal protein levels

**Figure 3:**
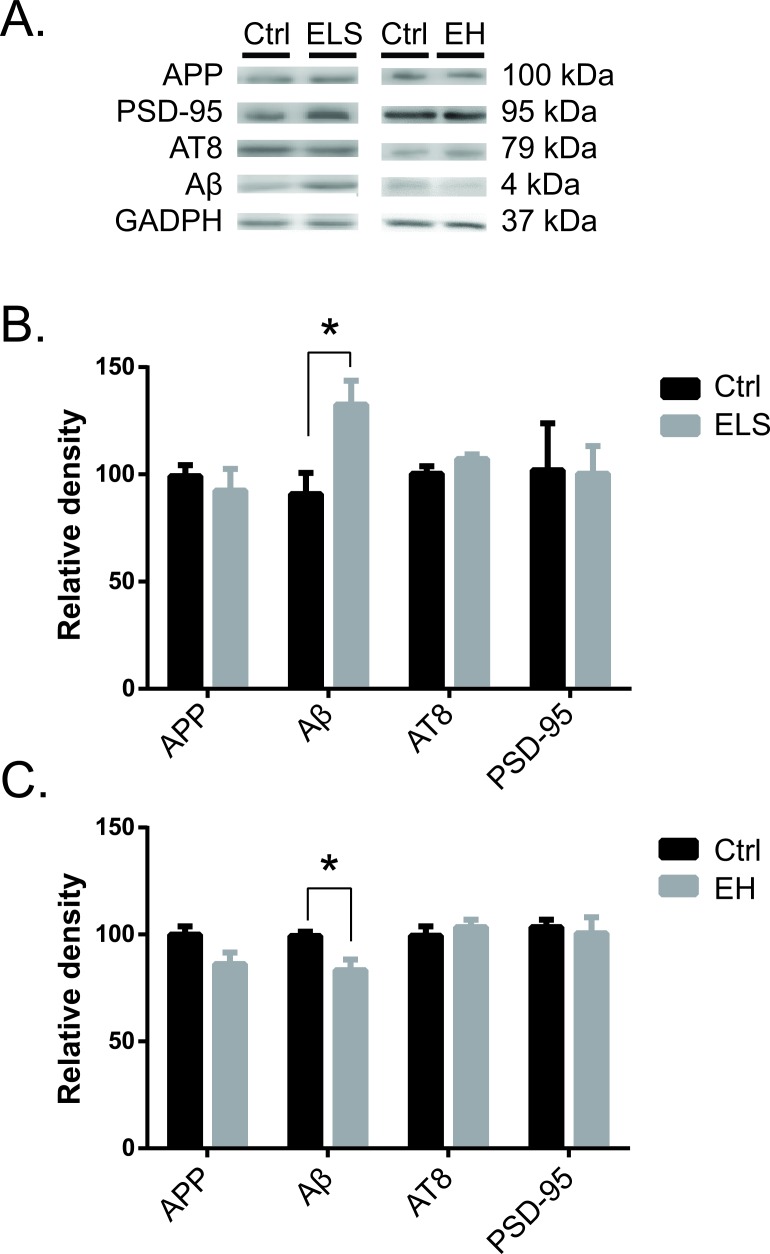
Early life experience alters hippocampal Aβ levels at PND 120 in biAT mice **A.** Western blot of hippocampal protein extracts demonstrated elevated Aβ levels after ELS **B.**, whereas EH led to a reduction in Aβ levels. **C.**. No effects of ELS or EH were observed on levels of APP. **B.** protein tau (AT8) **C.** or PSD-95 (H,I). ELS *vs* Ctrl, Ctrl: *n*= 7, ELS: *n* = 5; EH *vs* Ctrl, Ctrl: *n* = 3, EH: *n* = 5. Data are expressed as mean ± SEM.

To investigate whether ELS and EH modified AD related hallmarks differentially, we quantified protein Tau phosphorylated at serine 202 and threonine 205 (the epitope defined by Mab AT8), the levels of the amyloid peptides and their precursor APP. In addition, we measured the levels of PSD-95, a major post-synaptic protein (Figure 3A). Western blot analysis of hippocampal homogenates from the biAT mice revealed that ELS significantly increased in the levels of soluble monomeric Aβ (t(6) = −2.79, *p* < 0.05), while the levels of APP (t(10) = 0.65, ns), pTau at AT8 (t(9) = −1.38, ns) and PSD-95 levels (t(1.05) = 0.26, ns) were not altered (Figure 3B). In contrast, EH reduced levels of soluble, monomeric Aβ in the hippocampus (t(5) = 2.63, *p* < 0.05), while again the APP (t(6) = 1.80, ns), pTau at AT8 (t(4) = −0.72, ns) and PSD-95 levels (t(4) = 0.20, ns) were unaffected (Figure 3C).

### Anxiety and object recognition memory

#### Anxiety and locomotion

**Figure 4:**
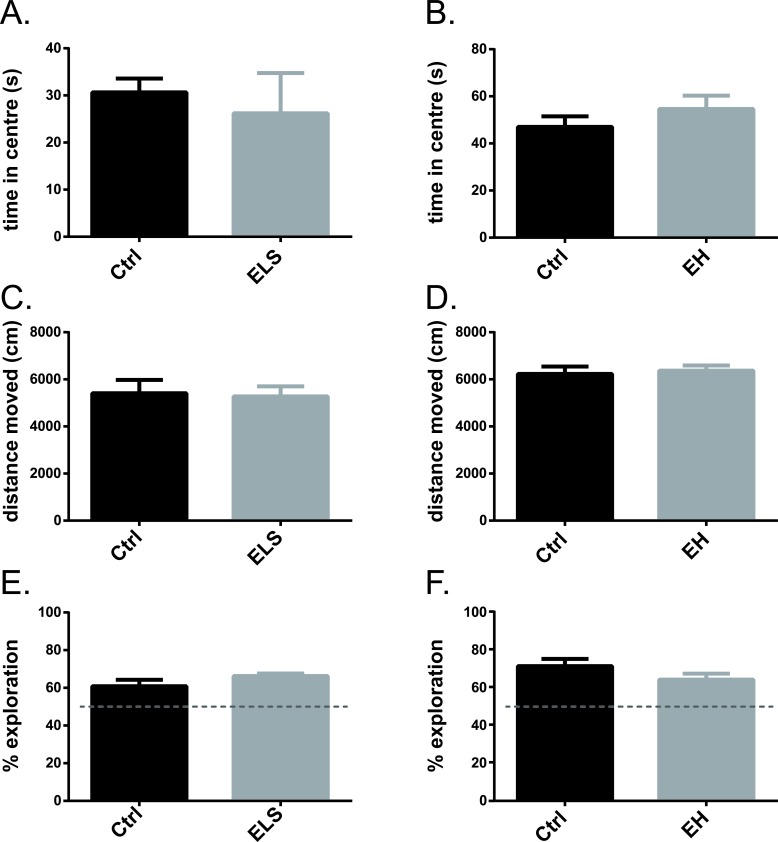
Explorative behaviour and cognition was not affected by early life experiences Time spent in the centre of the open field was not significantly different between ELS and Ctrl biAT (t(12) = 0.41, ns). **A.** or EH and Ctrl biAT mice (t(35) = −0.70, ns. **B.**. Total locomotor activity was also resistant to either ELS (Ctrl *vs* ELS: t(12) = 0.86, ns) **C.** or EH (Ctrl *vs* EH: t(35) = −0.38, ns). **D.**. Although all groups explored the novel object significantly above chance level (Ctrl *vs* ELS experiment: Ctrl: t(5) = 17.99, *p* < 0.000; ELS: t(5) = 3.16, *p* < 0.05; Ctrl *vs* EH experiment: Ctrl: t(15) = 2.96, *p* < 0.05; EH: t(19) = 4.60, *p* < 0.000) cognitive performance was comparable for all groups (Ctrl *vs* ELS: t(5.58) = 1.16, ns; Ctrl *vs* EH: t(34) = 0.43, ns). **E.**,**F.**. ELS *vs* Ctrl: Ctrl: *n* = 7, ELS: *n* = 7; EH *vs* Ctrl: Ctrl: *n* = 15, EH: *n* = 21. Data are expressed as mean ± SEM.

To investigate general locomotion and anxiety-like behaviour in young adult mice (PND 90 ± 3), the open field test was conducted. Exposure of biAT mice to ELS did not affect anxiety-like behaviour as the time spent in the centre zone of the arena was not different between ELS and control raised biAT mice (t(12) = 0.41, ns) (Figure 4A). In addition, exposure to ELS did not affect the general level of activity as no differences were detected in the total distance travelled throughout the arena (Ctrl *vs* ELS: t(12) = 0.86, ns) (Figure 4C). Likewise, in biAT mice EH had no effects on the time spent in the centre zone (t(35) = −0.70, ns) (Figure 4B), nor on the total distance travelled (t(35) = −0.38, ns) (Figure 4D).

#### Object recognition memory

In biAT mice, cognitive performance, as assessed by explorative behaviour in the novel object recognition test (PND 91 ± 3), was not affected by either ELS or EH (Figure 4E, 4F). When exposed to two identical objects during the training trial all animals expressed equal amounts of sniffing behaviour towards both objects, indicating no preference for the location of the object (data not shown). In the testing trial, biAT mice exposed to ELS and control rearing discriminated between the familiar and novel object, with exploration percentages being significantly higher than 50% (Ctrl: t(5) = 17.99, *p* < 0.000; ELS: t(5) = 3.16, *p* < 0.05). However, no difference in the amount of exploration was observed between ELS and control biAT mice (t(5.58) = 1.16, ns) (Figure 4E). Likewise, all EH and control biAT mice displayed novel object exploration levels that were significantly higher than 50% (Ctrl: t(15) = 2.96, *p* < 0.05; EH: t(19) = 4.60, *p* < 0.000). No difference in exploration was observed between any of the groups (t(34) = 0.43, ns) (Figure 4F).

### Dendritic morphology

#### Medial prefrontal cortex (mPFC)

**Figure 5:**
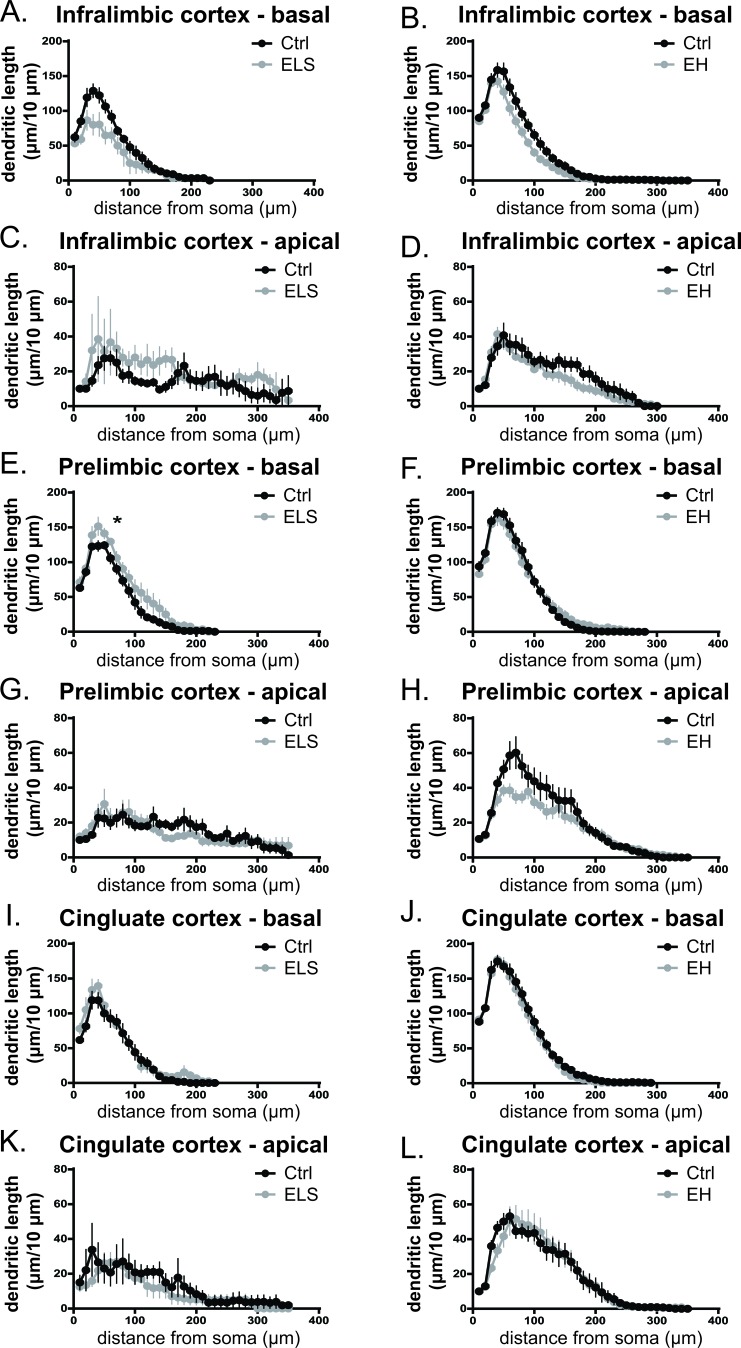
Dendritic morphology of mPFC subregions is differentially affected by ELS and EH Sholl plots of the distribution of apical and basal dendritic length at increasing distances from the centre of the cell body. In the basal dendrites of the infralimbic cortex, a trend towards a reduction in dendritic distribution was observed after both ELS and EH (ELS *vs* Ctrl: F(1,7) = 5.11, *p* = 0.058; EH *vs* Ctrl: F(1,17) = 4.31, *p* < 0.05). **A.**,**B.**. In the infralimbic cortex no effects of ELS or EH were reported on apical dendrites **C.**,**D.**. As a consequence of ELS, basal dendritic distribution of the prelimbic area was increased (F(1,11) = 4.92, *p* < 0.05), with significant post hoc differences at 60 μm from the centre of the cell body **E.**. No effects were observed in basal dendritic distribution in the prelimbic area after EH **F.**. The apical branches of the prelimbic cortex were not different between ELS and EH animals and their respective controls **G.**,**H.**. In the cingulate cortex, dendritic length at increasing distance from the centre of the cell body is comparable between ELS and Ctrl **I.**,**K.** and between EH and Ctrl **J.**,**L.** for both the apical and basal tree. 3-5 neurons from one animal were averaged (3-12 animals/group). Data are expressed as mean ± SEM.

We first examined dendritic morphology of neurons in the mPFC, which has been shown to be sensitive to early life adversity [26]. The mPFC is involved in executive functions and short-term memory, although subregions of the mPFC may respond differently as the infralimbic, prelimbic, and cingulate cortex are functionally and structurally distinct [27]. Indeed, the length of the basal dendritic tree of pyramidal neurons in the infralimbic cortex was reduced both as a consequence of ELS and EH in biAT mice compared to their respective control groups (ELS *vs* Ctrl: t(7) = 2.40, *p* < 0.05; EH *vs* Ctrl: t(17) = 2.42, *p* < 0.05) (Table 1). Furthermore, after ELS exposure the dendritic complexity index (DCI) (t(7) = 3.73, *p* < 0.05), and the number of branch points (t(7) = 2.56, *p* < 0.05) of the basal dendrites were significantly reduced in the infralimbic cortex. To further investigate the effects of early life environment on cellular morphology a segmental Sholl analysis was performed which examines changes in dendritic length as a function of radial distance from the soma. The reductions in basal branch length were, albeit mildly, reflected in the segmental distribution of the basal dendrites (Figure 5A, 5B) with a trend towards a reduction by both EH and ELS treatments compared to their respective controls (ELS *vs* Ctrl: F(1,7) = 5.11, *p* = 0.058; EH *vs* Ctrl: F(1,17) = 4.31, *p* < 0.05). No effect of either early life manipulation were reported on apical dendrites of the infralimbic cortex (Figure 5C, 5D).

**Table 1:** Morphological alterations of the dendritic tree of neurons of subregions of the medial prefrontal cortex after ELS or EH in adult mice (PND 120) Ctrl

			Ctrl	ELS	Ctrl	EH
**Infralimbic**	Basal	Branch length (μm)	916 ± 32	651 ± 126*	1253 ± 113	979 ± 73*
		DCI	5415 ± 264	2662 ± 963**	5769 ± 1017	4477 ± 419
		Branch points	6.14 ± 0.42	3.83 ± 1.01*	6.82 ± 0.80	6.28 ± 0.46
	Apical	Branch length (μm)	476 ± 93	656 ± 101	603 ± 85	478 ± 51
		DCI	7556 ± 1768	18410 ± 6607^$^	37806 ± 11205	14485 ± 2578
		Branch points	3.00 ± 0.32	3.50 ± 0.76	5.58 ± 0.72	4.62 ± 0.57
**Prelimbic**	Basal	Branch length (μm)	924 ± 66	1218 ± 118*	1397 ± 87	1251 ± 77
		DCI	4938 ± 428	9007 ± 2113*	8359 ± 1428	7854 ± 850
		Branch points	5.83 ± 0.35	7.9 ± 0.70*	8.22 ± 0.92	8.76 ± 0.54
	Apical	Branch length (μm)	469 ± 65	490 ± 60	733 ± 83	695 ± 56
		DCI	6848 ± 1726	13538 ± 4768	59072 ± 13985	56768 ± 11618
		Branch points	3.10 ± 0.65	3.1 ± 0.75	6.91 ± 0.69	6.91 ± 0.66
**Cingulate**	Basal	Branch length (μm)	855 ± 102	960 ± 66	1400 ± 72	1438 ± 111
		DCI	7317 ± 1941	4496 ± 667	8292 ± 719	8433 ± 1060
		Branch points	7.47 ± 1.12	6.63 ± 1.03	8.46 ± 0.50	8.85 ± 0.62
	Apical	Branch length (μm)	357 ± 69	316 ± 51	825 ± 53	665 ± 95
		DCI	3162 ± 1131	4256 ± 2075	70986 ± 16210	50530 ± 13664
		Branch points	2.47 ± 0.72	2.13 ± 0.66	7.91 ± 0.84	6.35 ± 1.08

Values are presented as mean ± SEM. Significance to its relative control group is indicated as follows:

** *p* < 0.01,

* *p* < 0.05,

$ *p* < 0.1.

In the basal tree of pyramidal neurons of the prelimbic cortex, ELS significantly increased the dendritic branch length (t(10) = −2.61, *p* < 0.05), the DCI (t(9) = −3.69, *p* < 0.05), and the number of branch points (t(9) = −4.61, *p* < 0.001) (Table 1). This increase was also observed in the dendritic Sholl distribution after ELS (F(1,11) = 4.92, *p* < 0.05) (Figure 5E). In contrast, EH did not affect the basal dendritic tree in the prelimbic cortex (Table 1, Figure 5F). In the apical tree of pyramidal neurons of the prelimbic cortex branch length, DCI, number of branch points (Table 1), and dendritic Sholl distribution (Figure 5G, 5H) were not significantly altered between ELS *versus* control or EH *versus* control biAT mice.

In the cingulate cortex, apical and basal branch length, DCI, and the number of branch points did not differ significantly between ELS *versus* control or EH *versus* control biAT mice (Table 1). Also Sholl analysis of the apical and basal dendrites revealed no significant differences in dendritic distribution in this subregion after any of the early life manipulations (Figure 5I-5L).

#### Amygdala

**Figure 6:**
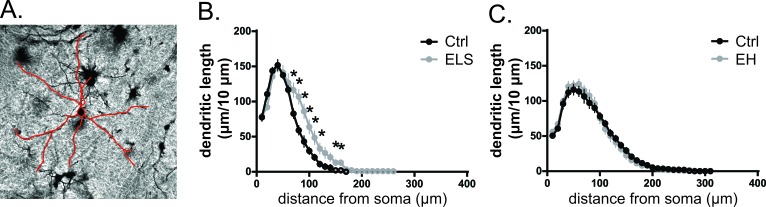
Early life stress increased neuronal complexity in the basolateral amygdala **A.** Representative image of the basolateral nucleus of the amygdala from Golgi-Cox-stained coronal brain section. **B.** ELS significantly increased the segmental complexity (F(1,7) = 6.69, *p* < 0.05) between 70 and 120 μm, and between 150 and 160 μm from the soma. **C.** Neuronal complexity was not affected by EH. Data of 3-5 neurons from one animal were averaged (3-12 animals/group) and expressed as mean ± SEM.

Neurons of the basolateral amygdala, involved in emotional processing, have been reported to undergo substantial dendritic remodelling following stress in adult animals [28]. Indeed, we observed that ELS increased the branch length of stellate neurons in the amygdala of biAT mice (t(7) = −2.72, *p* < 0.05) (Table 2), but it did not affect the number of branch points or the DCI. Sholl analysis revealed that the differences in branch length originated mainly from the distal portion of the dendritic tree (F(1,7) = 6.69, *p* < 0.05), which was more pronounced in ELS animals in the segments between 70 to 120 μm, and 150 to 160 μm from the soma, whereas no differences were observed in the proximal 60 μm from the soma (Figure 6A). EH did not affect dendritic morphology in the amygdala at all (Figure 6B).

**Table 2:** Morphological alterations of the dendritic tree of stellate neurons of the basolateral nucleus of the amygdala after ELS and EH in adult mice (PND 120)

		Ctrl	ELS	Ctrl	EH
**Amygdala**	Branch length (μm)	941 ± 42	1204 ± 139*	1203 ± 57	1200 ± 88
	DCI	5837 ± 667	9209 ± 3526	12300 ± 1244	11441 ± 2375
	Branch points	8.02 ± 0.28	8.78 ± 1.61	8.78 ± 0.69	8.41 ± 0.84

Values are presented as mean ± SEM. Significance to its relative control group is indicated as follows:

* *p* < 0.05.

#### CA1 and CA3 of the hippocampus

**Figure 7:**
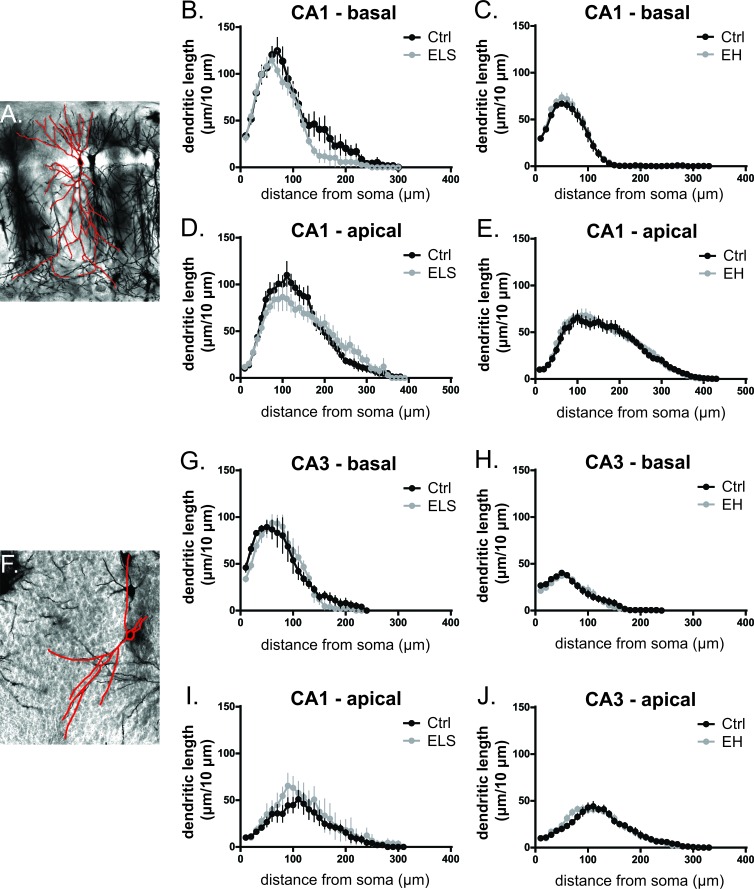
Neuronal morphology of CA1 and CA3 pyramidal neurons is unaffected by early life experiences Representative images from pyramidal neurons in CA1 **A.** and CA3 **F.** areas from Golgi-Cox staining of coronal sections. Sholl plots indicated that the distribution in CA1 of basal **B.**,**C.** and apical **D.**,**E.** dendritic length at increasing distances from the centre of the cell body is comparable between groups. In the CA3, the segmental distribution of basal branches of the neuron is not affected by ELS **G.** and EH **H.**. The segmental distribution of the apical branches is also comparable between ELS and Ctrl **I.** and EH and Ctrl **J.** animals. Data from 3-5 neurons from one animal were averaged (*n* = 5-12 animals/group) and expressed as mean ± SEM.

The sensitivity of the hippocampus, which is critically involved in learning and memory, of biAT mice to early life experiences was assessed in the CA1 and CA3 subregions (Figure 7A, 7F). We observed that in control and ELS-reared biAT mice, apical and basal branches of pyramidal neurons were comparable in length, in number of branch points and in DCI (Table 3). Likewise, EH did not alter hippocampal CA1 and CA3 apical and basal pyramidal dendritic morphology compared to control reared biAT mice (Table 3). Accordingly, Sholl analysis failed to reveal any effects of ELS or EH on dendritic parameters in the hippocampus of young adult biAT mice (Figure 7).

**Table 3:** Morphological alterations of the dendritic tree of hippocampal CA1 and CA3 pyramidal neurons after ELS or EH in adult mice (PND 120)

			Ctrl	ELS	Ctrl	EH
**CA1**	Basal	Branch length (μm)	1017 ± 94	945 ± 56	541 ± 58	574 ± 45
		DCI	25036 ± 3443	28390 ± 3429	6191 ± 1499	7061 ± 887
		Branch points	9.81 ± 0.85	9.85 ± 0.55	4.88 ± 0.51	5.48 ± 0.36
	Apical	Branch length (μm)	1557 ± 103	1440 ± 160	1239 ± 80	1380 ± 100
		DCI	442479 ± 69835	410175 ± 92073	198886 ± 35130	242883 ± 33503
		Branch points	17.19 ± 1.15	15.55 ± 1.95	13.01 ± 1.35	13.92 ± 1.12
**CA3**	Basal	Branch length (μm)	879 ± 128	821 ± 83	339 ± 39	323 ± 24
		DCI	8226 ± 2932	14579 ± 3586	1728 ± 316.69	1573 ± 305
		Branch points	5.61 ± 0.85	8.23 ± 1.58	1.96 ± 0.26	1.66 ± 0.19
	Apical	Branch length (μm)	614 ± 91	524 ± 30	551 ± 36	592 ± 39
		DCI	24140 ± 7983	13473 ± 429	12338 ± 2100	15090 ± 2286
		Branch points	4.97 ± 0.97	4.07 ± 0.22	3.60 ± 0.36	4.01 ± 0.32

Values are presented as mean ± SEM. Significance to its relative control group is indicated as follows

* *p* < 0.05.

## DISCUSSION

In order to define a possible link between early life experiences and the later development of AD, we investigated the effects of early life stress (ELS) and early handling (EH) from PND 2-9 on survival and life expectancy, on hippocampal Aβ levels, on memory, and on dendritic complexity in a bigenic mouse model of AD with transgenic co-expression of APP.V717I and Tau.P301L (biAT mice) [21]. We report that despite the strong genetic background in this model, ELS reduced long term survival and enhanced hippocampal Aβ levels at adulthood. By contrast, EH enhanced survival and reduced hippocampal Aβ levels. At PND 90, the cognition of these young adult biAT mice was not affected by ELS or EH. Dendritic complexity was slightly affected in the basal branches of the mPFC and in the amygdala, but not in the hippocampus.

### Experimental model

To examine the effect of the early life environment, pups were raised in an environment with limited nesting and bedding material from PND 2 to 9 (ELS), which induces stress in the dam and pups. In contrast, a similar group of biAT mice were exposed to daily handling for 15 minutes throughout the same period, early handling (EH), which is known to enhance maternal care upon reunion. The body weight gain of pups from PND 2-9 was significantly decreased by ELS while significantly increased by EH, although these differences were no longer evident at 4 months of age. The relatively mild manipulations from PND 2-9 resulted in a comparable phenotype as reported before, and validated the effectiveness of our experimental manipulation [22, 23].

We investigated transgenic mice with postnatal, neuron specific co-expression of human mutant APP.V717I and Tau.P301L protein [21]. Both transgenes are under control of the mouse thy1 gene promoter and become expressed in the 2^nd^ week postnatally, which coincided partially with the experimental PND 2-9 treatment period.

### Life expectancy

A key finding of our study was that different early life experiences differentially affected life expectancy of biAT mice. We report a profound negative modulatory effect of ELS on later survival, leading to a two-fold lower life expectancy of biAT mice subjected to ELS when compared to control biAT mice. In contrast, EH significantly increased the life expectancy of the biAT mice by 1.6-fold. The juvenile age during which the mortality rate increased in the ELS group suggests a disturbed or delayed neurodevelopmental defect, which apparently is prevented by EH.

Increased early mortality is a phenomenon which has previously been observed in genetic mouse models of AD, in particular in the more complex models. Previous studies in the current biAT mice and in the parental Tau.P301L mice with hindbrain tauopathy revealed an inverse relationship between brainstem tauopathy and lifespan [21, 29]. The brainstem controls autonomous vital systems such as breathing, swallowing, and blood pressure, suggesting that the premature death of Tau.P301L mice is caused by disturbances in these processes. However, throughout the 4 months of observation in the current study, no markedly higher number of deaths occurred in the littermates that expresses Tau.P301L only. This implies the additional and specific contribution of the APP.V717I mutant protein, possibly in combination with the tau mutation, in the early death of the biAT mice [21]. Evidence is accumulating that, similar to other models, young biAT mice are prone to epileptic activity which is also the cause of their premature death (HM, FVL, data not shown). Indeed, different transgenic AD mouse models that express mutant forms of APP were reported to die prematurely relative to wild type mice from the same strain [30–38]. Various studies demonstrated that premature death is closely associated with spontaneous seizures and abnormal epileptiform electroencephalography (EEG) activities [39, 40]. The implicated contribution of amyloid peptides and/or APP metabolites, alone and even more when combined with mutant protein tau [41], in inducing neuronal hyperexcitability and epilepsy susceptibility is evident. These animal model observations are consistent with the higher incidence of epileptic activity in aged AD patients as compared to non-dementing elderly [42].

Interestingly, ELS was reported in various models to elicit higher incidence of spontaneous seizures [43–46] and enhanced excitability [47, 48]. The combined data link ELS to the observed reduced survival in biAT animals by potentiating epileptic seizures, underlined additionally by the observed enhanced Aβ levels in the hippocampus of the ELS-reared biAT mice (see below). Interestingly, EH reverses this phenomenon and exerts protective effects against early mortality caused by seizure vulnerability. The combined data indicate that additional processes, besides regulation of seizure vulnerability, are modulated by positive early life experiences. Importantly, we observed that EH reduced the Aβ levels in the hippocampus, which may reduce the epileptic activity and thereby enhance life expectancy of biAT mice.

### Pathological biomarkers

Although amyloid plaques and neurofibrillary tauopathy develop from 8-10 months onwards in the current bigenic model of AD [21], we confirmed meaningful levels of soluble Aβ in the hippocampus of young adult biAT mice (4 months). In addition, phosphorylated protein tau was already evident at the same age in biAT mice. This combined biochemical and pathological profile warrants further investigations of early phase disease development, rather than end-stage disease consequences, which have been described most abundantly.

Interestingly, we observed that hippocampal soluble Aβ levels were further enhanced by ELS and reduced by EH in young adult biAT mice, 4 months after both the respective early life treatments. As hippocampal APP levels were unchanged, the ELS and EH treatments may affect APP-processing, respectively enhancing and inhibiting the amyloidogenic pathways. In this respect it is of interest that the promoter regions of the gene encoding β-site amyloid cleaving enzyme (BACE), which is responsible for the pathogenic cleavage of the APP protein, contains several glucocorticoid-response binding elements [49]. The activation by glucocorticoids released during stress could thereby increase amyloid peptide production in the biAT mice, and possibly in the brain of AD patients [50]. Thus, modulation of glucocorticoid levels by early life experiences could regulate BACE activity, thereby controlling post-translational processing of APP and the Aβ peptide levels.

Alternatively, the enzymes that are implicated in the clearance and degradation of amyloid peptides from the brain can be involved. For instance, insulin-degrading enzyme (IDE) which degrades Aβ, is claimed to be dysfunctional in AD and contributes to its pathogenesis [51, 52], while IDE activity is inhibited by glucocorticoids neprilysin, which rduces Aβ load, [53, 54]. Another Aβ degrading peptidase, is also regulated by glucocorticoids [55]. Finally, environmental enrichment, which is known to reduce Aβ load in animals [56, 57] and humans [58, 59], also influences neprilysin levels and activity [56]. It will be interesting to determine which of these mechanisms explains the observed differential effect of ELS and EH on the Aβ levels in the brain of young adult biAT mice.

### Behaviour and dendritic morphology

Previous studies in young adult biAT mice revealed impaired cognitive performance in the novel object recognition test, compared to wild type mice [21]. We confirm that also at 3 months of age, biAT mice successfully identified the novel object, while neither ELS nor EH modulated this capacity. Possibly, the profound effects of the transgenic background on cognition obscured any potential additional effects of ELS and EH. Alternatively, we analysed a mixed group of male and female mice, from which we know that females are more resistant to ELS than males [23]. Moreover, a potential bias could be have been introduced in our study by the fact that the most resilient animals have been studied as more vulnerable mice had already died before the test at 3 months of age. Especially in the ELS group over 60% of the original cohort could not be included in the analyses at adult age.

Following ELS, the dendritic morphology of the basolateral amygdala and prelimbic cortex was enhanced, which may be related to increased activity in these regions based on the expression of conditioned fear after early life adversity, as reported before [60–62]. In basal dendrites of the infralimbic mPFC neurons, we demonstrated a reduction in dendritic complexity both after ELS and EH. In line with our findings, the ELS paradigm reduced the dendritic complexity of the mPFC [63], although very little is known about effects of EH on dendritic morphology in the mPFC. In AD patients, activity in the mPFC is increased, possibly as a compensation for the decline in cognitive capacity in other brain areas [64]. While ELS decreased dendritic morphology of the mPFC, EH decreased pathological AD markers, which may make the compensatory overactivation of the mPFC no longer necessary, thereby potentially indirectly reducing dendritic morphology.

No major effects of ELS and EH were identified on the gross dendritic morphology in the CA1 and CA3 of the hippocampus, while also hippocampal PSD-95 levels were unchanged, indicating that hippocampal integrity was not further affected by ELS or EH in the biAT mice. Other studies have reported alterations in dendritic morphology in the hippocampus using the same stress paradigm [65], however, this has never before been studied in a complex transgenic AD model.

### Putative mechanism: HPA axis activity

Previous findings suggest that early life adversity can lead to persistently increased HPA axis reactivity and result in enhanced glucocorticoid secretion in response to stress [22, 66, 67]. In contrast, early life enhancement persistently attenuates the stress reactivity in the adult brain by dampening HPA axis activity, resulting in reduced glucocorticoid secretion in response to stress [24]. Furthermore, stress and elevated glucocorticoid levels have been reported to increase amyloid pathology and accelerate the development of NFT in an AD mouse model [50], and such effects could be rescued by blocking glucocorticoid receptors [68]. Although this needs experimental confirmation, these studies suggest that early life experiences can accelerate or delay the appearance of AD pathology in biAT mice, possibly *via* changes in HPA axis activity.

There is indeed ample evidence that the HPA axis is affected in patients suffering from AD, as reflected by markedly elevated basal levels of circulating cortisol, also in early stages of the disease [9, 69–78] and a failure to show cortisol suppression after a dexamethasone challenge [78–80]. Although AD patients show elevated basal cortisol levels, their HPA dysfunction did not worsen as the disease progressed, indicating that HPA axis dysfunction was mainly implicated in the early stages of the disease [9]. This suggests that in particular early alterations in HPA axis activity could contribute to the onset and possible acceleration of AD pathogenesis. Whether this is related to early life experiences remains to be investigated.

## CONCLUSION

We report here, to our knowledge for the that exposure to early life stress significantly decreases the life expectancy in biAT mice, parallel to enhanced soluble Aβ levels in the hippocampus. Conversely, early handling during the same period rather increased life expectancy and reduced soluble Aβ levels. Although the studied transgenic biAT mice by their very nature do not model sporadic AD, the results do underline the importance of a modulatory role of early life experiences, superimposed on a genetic prodromal background on relevant outcome parameters of AD. Following the early life manipulations, persistent alterations in the posttranslational processing of APP may occur. While this study supports a role for early alterations in HPA axis activity in the onset of AD pathogenesis, future experiments are required to identify underlying mechanisms which may help establish more directly the causal implications of early life experiences in AD aetiology.

## MATERIALS AND METHODS

### Animals

Bigenic APP.V717I x Tau.T301P (biAT) mice, and their Tau.P301L littermates, were bred in-house by crossing male heterozygous APP.V717I mice with homozygous Tau.P301L females, all in the FVB background [21]. One male animal was housed for 2 weeks with 2 female mice. At the beginning of the third gestational week, pregnant females were housed singly. All cages were covered with filter tops to prevent extra stress to the dams, and inspected daily between 7:00 and 10:00 AM. When a new born litter was encountered that day was assigned as postnatal day 0 (PND 0). The dams and litters were left undisturbed until PND 2 and kept under standard housing conditions (1 piece of nesting material, 12 hr light/dark cycle, lights on at 7:00 AM, humidity 40-60%, temperature 21±1°C) with unlimited access to food and water. All cages were also inspected daily for eventual deaths and the dates noted to draft the survival curves. In all groups, equal numbers of male and female mice were analysed. Animals were maintained and experiments were conducted in accordance with regulations of the KU Leuven and the European Community Council Directive (86/609/EC).

### Early life stress

We examined how early life stress (ELS) affects survival, AD pathology, behaviour and dendritic morphology in biAT mice. Chronic ELS was induced by housing dams with limited nesting and bedding material from PND 2 to 9 [22, 23]. Dams and their litters were weighed at PND 2 and randomly assigned to the ELS or control condition. In total 7 litters were assigned to each condition, resulting in a total of 32 control mice and 28 ELS mice. Control dams were provided with normal sawdust bedding and nesting material: a square piece of cotton 5 × 5 cm (Technilab-BMI, Someren, the Netherlands). The ELS dams were provided with a strongly reduced amount of sawdust bedding and half the nesting material (2.5 × 5 cm) and with a fine-gauge stainless steel mesh placed 1 cm above the cage floor. Both control and ELS cages were left undisturbed until the end of the ELS regime at PND 9. Then all mice were weighed and returned to standard cages, with normal amounts of sawdust bedding and nesting material until weaning at PND 21. Tail biopsies were collected from all offspring mice for genotyping by standard PCR analysis. All animals were then housed with 2-6 same sex littermates per cage. All experimental mice were left undisturbed, except for cage cleaning once a week, until behavioural testing.

### Early life handling

In a second series of experiments we examined how early handling (EH) affects survival, AD pathology, behaviour and dendritic morphology in biAT mice. Dams and their litters were weighed at PND 2 and randomly assigned to the EH or control condition. In total 5 litters were assigned to the control condition and 4 litters to the EH condition, resulting in 15 control mice and 23 EH mice. Control mice were housed with normal nesting and bedding material and were left undisturbed between PND 2-9. EH from PND 2-9 was induced by separating the dam and pups daily for 15 minutes between 9 AM and 11 AM. The dams and pups were placed in clean separate cages and reunited after 15 minutes in their home cage which was supplemented with 2 pieces of cotton nesting materials [24, 81]. During the separation, pups were placed on a heating pad at 32^°^C. On PND 9, all mice were weighed and placed in standard cages, with sufficient bedding and nesting material until weaning at PND 21. Upon weaning, tail biopsies were collected for genotyping, and mice were housed with 2-6 same sex littermates per cage. All experimental mice were left undisturbed, except for cage cleaning once a week, until testing.

### Behavioural testing

Open field: On PND 90±3, the open field test was conducted between 8-12 AM in an empty arena (50 × 50 x 50 cm) with black walls and a translucent floor, dimly lit from underneath. Number of mice analysed: ELS *vs* Ctrl each *n* = 7; EH *vs* Ctrl: *n* = 21 and *n* = 15 respectively. Animals were placed in a corner facing the wall, after which their exploration of the arena was monitored. The apparatus was virtually divided into an outer border and inner zone (30 × 30 cm) and the time and distance spent and travelled in each zone recorded, as well as the total time and distance each mice was mobile and travelled.

Object recognition: On PND 91±3, one day after the open field test, the mice were subjected to the novel object recognition task between 8 AM and 4 PM, in the same arena as the open field task. The number of mice analysed: ELS *vs* Ctrl, *n* = 7 each; EH *vs* Ctrl: *n* = 21 and *n* = 15, respectively. During the training, each mouse was granted 8 minutes to explore two identical objects (blue glass marbles, 5 cm diameter) placed equidistantly from the walls and each other. After 4 hours, the test trial was conducted using one original familiar object and one novel object (red plastic cube, 5 cm diameter) placed in exactly the same locations as during the training. Mice were reintroduced into the arena for 8 minutes to explore the novel and familiar object. The relative ratio of time spent on the novel object divided by total (novel + familiar) exploration time was used as the index. An index of over 50% preference for the novel object, was taken as recognition of the known object observed during the training trial. Mice that were immobile or spent less than 10 sec exploring the objects were excluded from the analysis.

### Western blot analysis

Western blot analysis (*n* = 4/group) was used to assess biochemical levels: amyloid peptides (6E10, Biolegend, lot no. 88718, 1:1000, 4 kDa), APP (6E10, Biolegend, lot no. 88718, 1:1000, 100 kDa), PSD-95 (D27E11, Cell Signalling, lot no. 23450S, 1:1000, 95 kDa), phosphorylated tau (AT8, Thermo Scientific, 1:200, 79 kDa), and GADPH (14C10, Cell Signalling, lot no. 36835, 1:3000, 37 kDa). Protein brain extracts were prepared following rapid decapitation from one hemisphere which was snap frozen and stored on −80°C until processing. Hippocampi were dissected and homogenised in RIPA buffer (150 mM NaCl, 1% NP-40, 0.5% sodium deoxycholate, 0.1% SDS, pH = 6.8) using small pellet mixers, then incubated for 10 min at room temperature and subsequently sonicated for 2×30 sec at maximum intensity, again incubated for 10 min and then centrifuged (1 min, 10000xg, 4°C). The supernatants were collected and the protein concentrations determined by a BCA Protein Assay (Pierce, The Netherlands). An aliquot equivalent to 15 μg protein was separated by electrophoresis on 12.5% polyacrylamide-SDS gels with 5% stacking gels and proteins transferred to PVDF membranes for 2 hours at 75 V in Towbin buffer (25 mM Tris, 192 mM glycine, 20% methanol, pH = 8.3). The membranes were blocked with TBST (TBS + 0.1% Tween-20) containing 5% BSA for 1 hour, rinsed in TBST and strips were incubated with primary antibody overnight at 4°C. Blots were washed with TBST and incubated for 2 hours with secondary antibody. After thorough washing with TBS, signal was developed (Licor Odyssey FC; Leusden, the Netherlands). Signal intensities were measured using dedicated software (ImageJ; NIH; Bethesda) and normalised against GADPH as internal marker. Individual protein levels were calculated as the mean of 3 independent replications.

### Dendritic morphology

Golgi-Cox impregnation was performed as described previously [82]. Immediately after decapitation, one hemisphere was immersed in Golgi-Cox solution (5% K2CrO4, 5% HgCL and 5% K2Cr2O7) for 14 days, after which they were embedded in celloidine and cut coronally into 200 μm thick sections. The dendritic tree of neurons in the amygdala (bregma −2.0 mm to −3.2 mm), the CA1 and CA3 area of the hippocampus (bregma −2.0 mm to −3.2 mm), and the IL, PRL, and CG of the mPFC (bregma 2.2 mm to 4.2 mm) were analysed by obtaining Z-stacks (step-size 1 μm) using a microscope (LSM510, Zeiss, Germany) with a 20x magnification. Cells were reconstructed using dedicated software (Image Pro Analysis and Neurodraw reconstruction). Neurons were included according to criteria as previously described [28, 83]: (1) the presence of untruncated dendrites, (2) consistent and dark impregnation along the entire extent of all dendrites, and (3) relative isolation from neighbouring impregnated neurons. A total of 3-5 neurons from each animal were averaged (3-12 animals/group). Structural measures included total branch length, number of branch points, and dendritic complexity index (DCI). The DCI was calculated by the formula: (∑ branchtip orders + # of branch tips)/(# of primary dendrites) x (total arbour length). In addition, for each reconstructed neuron, the 3D Sholl analysis was performed using dedicated software (NeuronStudio) [84].

### Statistical analysis

Statistical analysis was performed using SPSS 21.0 except for outlier analysis, which was conducted using Grubb's test (Graphpad Prism 5). All data are presented as mean ± SEM, with *p* < 0.05 considered statistically significant. All experimental groups were compared to their respective control group.

Analysis of mouse survival was conducted using the log rank test. Body weight and behaviour were analysed using an independent samples *t*-test, or by the non-parametric equivalent Mann Whitney U test if one of its assumptions was violated. Data from the novel object recognition test were analysed by one-sample *t*-test to compare the exploration percentage to 50 (no discrimination). For the analysis of the dendritic morphology, 3 to 5 neurons from one animal were averaged and compared using independent samples *t*-test. Statistical significances for segmental dendritic plots (Sholl analysis) were obtained from repeated measures ANOVA with adequate corrections (Greenhouse-Geisser) on the significant values when the sphericity assumption was not met, and post hoc *t*-test was conducted to determine the distance at which differences occurred.
